# Age- and Influenza Activity-Stratified Case Definitions of Influenza-Like Illness: Experience from Hospital-Based Influenza Surveillance in South Korea

**DOI:** 10.1371/journal.pone.0084873

**Published:** 2014-01-24

**Authors:** Tae Un Yang, Hee Jin Cheong, Joon Young Song, Jin Soo Lee, Seong-Heon Wie, Young Keun Kim, Won Suk Choi, Jacob Lee, Hye Won Jeong, Woo Joo Kim

**Affiliations:** 1 Division of Infectious Diseases, Department of Internal Medicine, Korea University College of Medicine, Seoul, Korea; 2 Asian Pacific Influenza Institute (APII), Seoul, Korea; 3 Division of Infectious Diseases, Department of Internal Medicine, Inha University School of Medicine, Incheon, Korea; 4 Division of Infectious Diseases, Department of Internal Medicine, Catholic University Medical College, St. Vincent's Hospital, Suwon, Korea; 5 Division of Infectious Diseases, Department of Internal Medicine, Yonsei University, Wonju College of Medicine, Wonju, Korea; 6 Division of Infectious Diseases, Department of Internal Medicine, Hallym University College of Medicine, Chuncheon, Korea; 7 Division of Infectious Diseases, Department of Internal Medicine, Chungbuk National University College of Medicine, Cheongju, Korea; University of Hong Kong, Hong Kong

## Abstract

**Objectives:**

This study aims to identify clinical case definitions of influenza with higher accuracy in patients stratified by age group and influenza activity using hospital-based surveillance system.

**Methods:**

In seven tertiary hospitals across South Korea during 2011–2012 influenza season, respiratory specimens were obtained from patients presenting an influenza-like illness (ILI), defined as having fever plus at least one of following symptoms: cough, sore throat or rhinorrhea. Influenza was confirmed by reverse transcriptase-polymerase chain reaction. We performed multivariate logistic regression analyses to identify clinical variables with better relation with laboratory-confirmed influenza, and compared the accuracy of combinations.

**Results:**

Over the study period, we enrolled 1417 patients, of which 647 had laboratory-confirmed influenza. Patients with cough, rhinorrhea, sore throat or headache were more likely to have influenza (*p*<0.05). The most accurate criterion across the study population was the combination of cough, rhinorrhea, sore throat and headache (sensitivity 71.3%, specificity 60.1% and AUROC 0.66). The combination of rhinorrhea, sore throat and sputum during the peak influenza activity period in the young age group showed higher accuracy than that using the whole population (sensitivity 89.3%, specificity 72.1%, and AUROC 0.81).

**Conclusions:**

The accuracy of clinical case definitions of influenza differed across age groups and influenza activity periods. Categorizing the entire population into subgroups would improve the detection of influenza patients in the hospital-based surveillance system.

## Introduction

World Health Organization (WHO) records indicate that, worldwide, influenza infection causes three to five million cases of severe illness and accounts for 250,000 to 500,000 deaths each year [1]. In South Korea, it is estimated that the annual prevalence of influenza is around 20%, and that influenza-related complications are attributed to 2300 deaths annually [2], [3]. Even setting the 2009 pandemic influenza A/H1N1 (2009 pdm H1N1) aside, influenza poses a substantial threat to public health and is associated with high morbidity and mortality.

The prompt diagnosis of influenza and early initiation of antiviral therapy may alleviate clinical symptoms, attenuate complications and reduce transmission. Additionally, a swift diagnosis can minimize the use of inappropriate antibiotic therapy. Influenza infection can be diagnosed with either a rapid influenza antigen test (RIAT) or reverse transcriptase polymerase chain reaction (RT-PCR). Because these confirmatory tests are not always available and require significant time to perform, they are not always performed in the context of an influenza outbreak. Furthermore, case definitions of influenza-like illness (ILI), in which patients exhibit several symptoms of influenza, have been defined in limited studies. Some clinical trials have attempted to utilize specific symptoms or signs to differentiate between ILIs and identify influenza infections among cases of ILI, but no single clinical finding has been consistently accurate enough to inform the clinical decision to treat potential cases with antiviral agents or not [4], [5].

From a public health perspective, clinical criteria that distinguish patients with higher probabilities of having influenza infections are important. Accurate clinical case definitions will provide increased opportunities for appropriate management of individual patients and reinforcement of influenza sentinel surveillance. However, clinical case definitions that are both highly sensitive and highly specific are difficult to obtain [4]. Therefore, influenza case definitions may vary depending on the purpose and scope of the analysis. For example, a case definition used in a nationwide or worldwide surveillance study may not be intended to capture all influenza cases, but to describe trends over time and estimate the overall impact of an epidemic [6]. In this case, considering the cost and effectiveness, it is necessary to use one simple case definition to monitor the trend worldwide. In contrast, physicians who are faced with patients presenting acute respiratory illness may require criteria that show higher accuracy in their management of individual cases.

It is important to increase the accuracy of influenza diagnoses by identifying and categorizing subgroups to improve the surveillance system as well as to help support proper case management. In this study, using surveillance data of Hospital-based Influenza Morbidity and Mortality (HIMM) in an emergency department (ED) setting, we aim to identify the best case definition composed of clinical symptoms and signs [7]. We applied several case definitions to patients who visited hospital ED with fever and respiratory symptoms and tested the sensitivity and specificity of the case definitions in identifying laboratory-confirmed influenza patients across different age groups and levels of influenza activity.

## Methods

### Ethics Statement

This research plan was approved by the Institutional Review Board (IRB) of each participating hospital (approval number): Korea University Guro Hospital (KUGH11088, KUGH12007-001), Korea University Ansan Hospital (AS11047), Inha University Hospital (11-1534), The Catholic University St. Vincent's Hospital (VC11ONME0118), Yonsei University Wonju Christian Hospital (CR311025), Hallym University Kangnam Sacred Heart Hospital (2011-06-50), and Chungbuk National University Hospital (2011-06-044). Written informed consent was obtained from all patients.

### Study Design

This study was conducted prospectively during the 2011–2012 influenza season (September 2011 through May 2012) as a part of hospital-based influenza active surveillance study. We collected data from patients who visited the emergency rooms (ER) of any of seven tertiary teaching hospitals in South Korea: Korea University Guro Hospital, Korea University Ansan Hospital, Hallym University Kangnam Sacred Heart Hospital, Inha University Hospital, Chungbuk National University Hospital, Saint Vincent Hospital of Catholic University or Wonju Christian Hospital of Yonsei University. The study population was restricted to persons aged 18 years or older who presented influenza-like illness. The ILI definition used in HIMM surveillance system (HIMM-ILI) was: presence of (1) body temperature ≥38°C and (2) at least one of the following three symptoms: cough, sore throat or rhinorrhea, within seven days prior to visiting the ER [7].

ER physicians were instructed to record clinical manifestations using a structured case report form (CRF) and to collect respiratory specimens from patients presenting with HIMM-ILI. Clinical and demographic data were collected by well-trained clinical research coordinators. The following parameters were included in the CRF: symptoms or signs (fever, chills, cough, sputum, sore throat, rhinorrhea, chest pain, dyspnea, diarrhea, nausea/vomiting, abdominal pain, headache, myalgia, wheezing, crackle, general weakness and seizure) and demographic data (gender, age, date of visiting hospital, influenza vaccination history and smoking history).

In this study, we evaluated the diagnostic accuracy of a multifaceted definition of influenza, using a combination of symptoms and signs, in patients visiting the ER. The diagnostic accuracy was further analyzed by subgrouping based on patients' ages and influenza activity. The study population was grouped into three age categories: young (aged 18–29 years), middle (aged 30–64 years) and old (aged ≥65 years). Levels of influenza activity in this study were defined using Korean Influenza Surveillance System (KISS) data from the 2011–2012 season. The KISS-ILI definition used was: presence of (1) body temperature ≥38°C and (2) cough or rhinorrhea. The epidemic threshold for the 2011–2012 influenza season in Korea was 3.8 KISS-ILI patients out of 1000 patients, and the highest KISS-ILI rate was 23.10. The epidemic period of the 2011–2012 influenza season of South Korea was from December 25, 2011 (week 53) through May 5, 2012 (week 18). Within the influenza epidemic period, influenza activity was classified into low, high and peak activity periods. We defined a ‘low influenza activity period’ as the time when the KISS-ILI rate is lower than 50th percentile between the threshold and the highest activity. A ‘high influenza activity period’ was defined as a time when KISS-ILI rate was higher than the 50th percentile. The ‘peak influenza activity period’ was defined as 5 weeks of the highest KISS-ILI rate in 2011–2012 season.

### Laboratory Confirmation

Influenza RT-PCR (Seeplex® Influenza A/B One Step Typing, Seegene, Inc., Korea) was performed for all respiratory specimens of each enrolled subject, and laboratory-confirmed influenza was defined as a case in which a sample displayed a positive RT-PCR result. Nasopharyngeal swabs were obtained using Copan Flocked Swabs. Following collection, respiratory specimens were immediately placed into vials containing 2–3 mL of virus transport medium. All inoculated vials were kept at 4°C until transported, and specimens were stored at ≤−70°C until testing.

### Statistical Analyses

Statistical analyses were performed using SAS software version 9.2 (SAS Institute, Cary, NC, USA) and MedCalc for Windows, version 12.3.0 (MedCalc Software, Mariakerke, Belgium). Student's *t* test was used for comparison of continuous variables and a χ^2^ test was used for categorical data. Variables found to be statistically significant in univariate analyses were entered into multivariate analysis using a logistic regression model to identify independent risk factors for a diagnosis of laboratory-confirmed influenza. Since fever was a part of the inclusion criteria of this study, it was not analyzed as an independent variable. All statistical tests were two-tailed, and a value of *p*<0.05 was considered statistically significant.

We compared combinations of symptoms for their accuracy in the identification of laboratory-confirmed influenza. Among variables from multivariate analysis, variables with the highest odds ratios were selected and combinations of those variables were evaluated for accuracy in detecting patients with influenza in different age groups and influenza activity periods. Diagnostic accuracy was assessed by calculating five test performance parameters - sensitivity, specificity, area under receiving operating curve (AUROC), positive likelihood ratio and negative likelihood ratio of each combination with 95 percent confidence intervals (CI). In the analysis, sensitivity was defined as the probability of having the case definition in a case of laboratory-confirmed influenza; specificity was defined as the probability of not having the case definition when the patient does not have laboratory-confirmed influenza. Higher AUROCs suggest the variables are better tools for identifying laboratory-confirmed influenza patients because they take both sensitivity and specificity into account.

## Results

### Demographic and Clinical Characteristics

Overall, we enrolled 1417 patients that met the inclusion criteria, among which 647 (45.7%) patients had a laboratory-confirmed diagnosis of influenza. Of those who visited the ER during the epidemic period (1310 patients), 48.9% (640 patients) were positive for influenza, and 6.5% (7 among 107 patients) were positive for influenza during the non-epidemic period. The proportion of laboratory-confirmed influenza among ER-visiting HIMM-ILI patients was highest among the middle age group (48.7%) and lowest among the young age group (40.7%).

The mean age of enrolled patients was 46.3±19.1 years old. There was no significant difference in age, gender, or vaccination status between those patients with laboratory-confirmed influenza and those whose laboratory tests for influenza were negative (*p*>0.05). With respect to influenza type/subtype, frequencies of influenza A, B and A/B coinfection were 72.5%, 24.6%, and 2.9%, respectively (Table 1). Influenza A was predominant compared to influenza B in both the younger (18–64 years) and older (≥65 years) age groups. However, the proportion of influenza A was higher in the older age group (38.0%, 122 among 321 patients) than in the younger age group (31.6%, 347 among 1096 patients) (*p*<0.05).

**Table 1:** Characteristics of patients with influenza-like illness and influenza types among patients with laboratory-confirmed influenza.

	Patients with laboratory-	Patients who tested	
	confirmed influenza	negative for influenza	
	(N = 647)	(N = 770)	*p-value*
**Demographic data and clinical manifestations**			
Age in years: mean (SD)	46.2 (18.8)	46.4 (19.4)	0.847
Age distribution: N (%)			
18 to 29 years	125 (19.3%)	182 (23.6%)	0.049
30 to 64 years	384 (59.4%)	405 (52.6%)	0.011
65+ years	138 (21.3%)	183 (23.8%)	0.275
Male gender: N (%)	254 (39.3%)	346 (44.9%)	0.031
Influenza vaccination status: N (%)†	155/605 (25.6%)	168/718 (23.4%)	0.349
Fever: °C (SD)	38.63 (0.54)	38.65 (0.60)	0.523
Time from ER visit to symptom onset: days (SD)	1.71 (1.65)	1.78 (1.82)	0.469
Symptoms and signs, N (%)			
Headache	332 (51.3%)	298 (38.7%)	<0.001
Myalgia	381 (58.9%)	363 (47.1%)	<0.001
Cough	592 (91.5%)	620 (80.5%)	<0.001
Sputum	472 (73.0%)	456 (59.2%)	<0.001
Sore throat	412 (63.7%)	375 (48.7%)	<0.001
Rhinorrhea	448 (69.2%)	354 (46.0%)	<0.001
Dyspnea	104 (16.1%)	137 (17.8%)	0.391
Chest pain	96 (14.8%)	82 (10.6%)	0.018
Nausea/vomiting	124 (19.2%)	144 (18.7%)	0.824
Abdominal discomfort	58 (9.0%)	79 (10.3%)	0.411
Diarrhea	54 (8.3%)	75 (9.7%)	0.364
Wheezing	30 (4.6%)	18 (2.3%)	0.017
Crackle	25 (3.9%)	24 (3.1%)	0.443
**Influenza type: N (%)**			
A	469 (72.5%)	-‡	
B	159 (24.6%)	-	
Both A&B	19 (2.9%)	-	

† Data of vaccination status were available in only 1323 out of 1417 patients.

‡ Not applicable.

SD, Standard deviation.

Data describing underlying comorbidities were available from one of the seven study hospitals (*n* = 305). In the analysis of these data, the underlying comorbidities were not significantly different between laboratory-confirmed influenza and laboratory test-negative HIMM-ILI patients.

In the analysis of clinical manifestations, the most frequently reported symptoms were similar between patients whose tests showed laboratory-confirmed influenza and HIMM-ILI patients whose influenza tests were negative. However, individuals with laboratory-confirmed influenza were more likely to have chills, general weakness, headache, myalgia, cough, sputum, sore throat, rhinorrhea, chest pain and wheezing compared to those without laboratory-confirmed influenza (*p*<0.05) (Table 1). To have fever ≥39°C (n = 399) did not aid in identifying laboratory-confirmed influenza (*p* = 0.620), and neither did fever ≥40°C (n = 44) (*p* = 0.61).

### Multivariate Analysis for ILI Case Definitions

Multivariate logistic regression analysis revealed a significant association between laboratory-confirmed influenza diagnoses and some clinical symptoms: cough (Odds ratio [OR] 2.40, 95% CI 1.68–3.42), rhinorrhea (OR 2.14, 95% CI 1.69–2.70), sore throat (OR 1.62, 95% CI 1.28–2.04) and headache (OR 1.34, 95% CI 1.05–1.70) (Table 2). We evaluated various combinations of cough, rhinorrhea, sore throat and headache for their abilities to distinguish influenza from non-influenza ILI using AUROC. Table 3 shows the five best-performing clinical case criteria for identification of influenza among patients in a whole population with HIMM-ILI. The highest AUROC value was found for the combination of cough, rhinorrhea, sore throat and headache (sensitivity 71.3%, specificity 60.1%, AUROC 0.66, positive likelihood ratio 1.8 and negative likelihood ratio 0.5).

**Table 2:** Univariate and multivariate analyses for clinical variables of laboratory-confirmed influenza.

	Univariate analysis			Multivariate analysis		
	Crude OR	(95% CI)	*p*-value	Adjusted OR	(95% CI)	*p*-value
**Constitutional symptoms**						
Chills	1.57	1.25–1.98	<0.001	1.06	0.82–1.38	0.647
General weakness	1.54	1.25–1.90	<0.001	1.11	0.86–1.42	0.425
Headache	1.67	1.35–2.06	<0.001	1.34	1.05–1.70	0.018
Myalgia	1.61	1.30–1.98	<0.001	1.21	0.95–1.55	0.126
**Respiratory symptoms**						
Cough	2.60	1.87–3.62	<0.001	2.40	1.68–3.42	<0.001
Sputum	1.86	1.48–2.33	<0.001	1.25	0.97–1.61	0.088
Sore throat	1.85	1.49–2.29	<0.001	1.62	1.28–2.04	<0.001
Rhinorrhea	2.65	2.13–3.29	<0.001	2.14	1.69–2.70	<0.001
Dyspnea	0.89	0.67–1.17	0.391	-†	-	-
Chest pain	1.46	1.07–2.00	0.018	1.02	0.73–1.44	0.906
**Physical findings**						
Wheezing	2.03	1.12–3.68	0.017	1.86	0.99–3.49	0.055
Crackle	1.25	0.71–2.21	0.443	-	-	-
**Other symptoms**						
Diarrhea	0.84	0.59–1.22	0.364	-	-	-
Nausea/vomiting	1.03	0.79–1.35	0.82	-	-	-
Abdominal discomfort	0.86	0.60–1.23	0.41	-	-	-

CI, Confidence interval.

† Indicates the variable was not entered in logistic regression analysis as it was not significant in univariate analysis.

**Table 3:** Accuracy of diverse influenza-like illness case definitions in identification of laboratory-confirmed influenza.

	Sensitivity (%)	Specificity (%)	AUROC	Positive LR	Negative LR
	(95% CI)	(95% CI)	(95% CI)	(95% CI)	(95% CI)
Cough, rhinorrhea, sore throat and headache	71.3 (65.4–76.7)	60.1 (57.2–63.0)	0.66 (0.63–0.68)	1.8 (1.6–2.0)	0.5 (0.4–0.6)
Rhinorrhea, sore throat and headache	69.9 (64.1–75.3)	60.2 (57.3–63.1)	0.65 (0.63–0.68)	1.8 (1.6–2.0)	0.5 (0.4–0.6)
Cough, rhinorrhea and sore throat	65.0 (60.4–69.4)	63.5 (60.4–66.6)	0.64 (0.62–0.67)	1.8 (1.6–2.0)	0.6 (0.5–0.6)
Cough, rhinorrhea and headache	66.7 (61.5–71.5)	61.5 (58.5–64.4)	0.64 (0.62–0.67)	1.7 (1.6–1.9)	0.5 (0.5–0.6)
Rhinorrhea and sore throat	63.3 (58.8–67.5)	63.7 (60.5–66.7)	0.64 (0.61–0.66)	1.7 (1.6–1.9)	0.6 (0.5–0.7)

Positive LR, Positive likelihood ratio; Negative LR, Negative likelihood ratio.

### Subgroup Analysis of ILI Case Definitions Relating to Age Group and Level of Influenza Activity

Multiple logistic regression analysis revealed significant associations of a positive test for influenza with rhinorrhea, cough, sore throat, and sputum in the young age group; cough, rhinorrhea, sore throat, headache, and myalgia in middle age group; and wheezing, diarrhea and rhinorrhea in old age group (*p*<0.05) (Table 4).

**Table 4:** Subgroup analysis of clinical variables for laboratory-confirmed influenza, stratified by age and influenza activity: multivariate analysis (odds ratios (95% CI)).

Clinical variables	Chills	Cough	Sputum	Sore throat	Rhinorrhea	Chest pain	Dyspnea	Diarrhea	Nausea/vomiting	Abdominal pain	Headache	Myalgia	Wheezing	General weakness	Crackle
**Age groups**															
Aged 18–29 years	-†	2.47*	1.86*	2.07*	4.35*	-	-	-	-	0.39*	-	-	-	-	-
(*n* = 307)		(1.17–5.24)	(1.03–3.33)	(1.20–3.56)	(2.39–7.92)					(0.17–0.90)					
Aged 30–64 years	1.12	2.84*	1.23	1.60*	1.88*	-	-	-	-	-	1.59*	1.46*	-	1.46	-
(*n* = 789)	(0.79–1.61)	(1.74–4.64)	(0.88–1.72)	(1.16–2.20)	(1.37–2.58)						(1.15–2.18)	(1.04–2.05)		(0.86–1.71)	
Aged 65+ years	-	-	-	1.61	1.86*	-	0.40*	3.44*	-	-	-	-	4.22*	-	-
(*n* = 321)				(0.97–2.67)	(1.16–2.99)		(0.23–0.72)	(1.05–11.32)					(1.60–11.14)		
**Levels of influenza activity**															
Epidemic period	1.09	2.30*	1.29	1.60*	2.11*	1.05	-	-	-	-	1.37*	1.23	1.81	1.11	-
(*n* = 1310)	(0.83–1.43)	(1.60–3.32)	(1.00–1.68)	(1.26–2.03)	(1.66–2.70)	(0.73–1.49)					(1.07–1.76)	(0.95–1.58)	(0.94–3.48)	(0.86–1.44)	
Low	-	5.18*	-	1.75	1.35	-	-	-	-	-	1.84*	-	-	-	-
(*n* = 216)		(1.87–14.35)		(0.97–3.18)	(0.74–2.47)						(1.02–3.35)				
High	1.09	1.92*	1.44*	1.57*	2.35*	1.27	-	-	-	-	1.20	1.20	1.95	1.24	-
(*n* = 1094)	(0.81–1.47)	(1.28–2.87)	(1.08–1.91)	(1.20–2.05)	(1.80–3.07)	(0.97–1.68)					(0.91–1.59)	(0.91–1.59)	(0.96–3.95)	(0.93–1.63)	
Peak	1.28	2.26*	1.42	1.91*	2.44*	-	-	-	-	0.45*	1.49	1.20	3.17	1.28	-
(*n* = 457)	(0.80–2.04)	(1.17–4.37)	(0.89–2.25)	(1.25–2.91)	(1.60–3.73)					(0.22–0.91)	(0.94–2.37)	(0.77–1.87)	(0.93–10.83)	(0.82–2.01)	

† Indicates that the variable was not entered in logistic regression analysis as it was not significant in univariate analysis.

* Indicates that the *p*-value of the data <0.05 in logistic regression analysis.

Note: Epidemic period (25 December 2011 [week 53]–5 May 2012 [week 18]) (*n* = 1811), non-epidemic period (25 September 2011 [week 40]–24 December 2011 [week 52], 6 May 2012 [week 19]–2 July 2012 [week 22]) (*n* = 147).

During the epidemic period, cough, rhinorrhea, sore throat and headache appeared to be associated with influenza infection, but none of these symptoms or signs aided in identification of influenza during the non-epidemic periods (Table 4). In the young age group, multivariate analysis showed that respiratory symptoms, such as cough, rhinorrhea, sputum and sore throat were associated with influenza regardless of the level of influenza activity. However, middle age patients with headache, myalgia or general weakness, along with respiratory symptoms, were more likely to be laboratory-confirmed influenza cases during the higher influenza activity period (data not shown).

### Combinations of Symptoms and Signs Having Improved Accuracy in Identification of Influenza Infection

We compared combinations of symptoms constructed from the variables identified from logistic regression analysis. In the young age group, the combination of rhinorrhea and cough had the highest AUROC (0.71), with a sensitivity of 60.7% and a specificity of 81.9%. In the middle age group, cough, rhinorrhea and headache showed the highest AUROC (0.66), with a sensitivity of 71.0% and specificity of 60.6%. In the old age group, the combination of rhinorrhea and wheezing had the highest AUROC (0.61), but the value was relatively lower than that seen in the younger age groups (data not shown).

Table 5 shows the most accurate clinical case definitions in each group as identified from combinations of symptoms identified in multivariate analysis. Each criterion, consisting of one to four variables, was evaluated for its accuracy in detecting influenza infection according to age group and level of influenza activity. Theoretically, there were 591 possible combinations according to the statistically significant variables in Table 4 in each group, stratified by age and influenza activity. However, as variables with higher OR were expected to show better accuracy, we combined the variable with the highest OR and three or less variables with the next highest OR and the criteria were evaluated for accuracy in each of the categories. Among the 76 combination we tested, the highest AUROC values were seen during the peak influenza activity period and in the young age group which suggests the case definitions were more accurate in the younger age group during periods of higher influenza activity. The accuracy of the combination of rhinorrhea, sore throat and sputum during the peak influenza activity period in the young age group showed a sensitivity of 89.3%, a specificity of 72.1% and an AUROC of 0.81.

**Table 5:** Accuracy of diverse influenza-like illness symptom combinations, stratified by age and influenza activity.

Age group	Influenza activity		Sensitivity (%)	Specificity (%)	AUROC	Positive LR	Negative LR
			(95% CI)	(95% CI)		(95% CI)	(95% CI)
**Young**	**Peak**	Rhinorrhea, sore throat and sputum	89.3 (71.8–97.7)	72.1 (59.2–82.9)	0.81 (0.71–0.88)	3.2 (2.1–4.9)	0.2 (0.0–0.4)
	**High**	Rhinorrhea, sore throat and sputum	74.7 (62.9–84.2)	68.1 (60.4–75.2)	0.71 (0.65–0.77)	2.3 (1.8–3.0)	0.4 (0.2–0.6)
	**Low**	Cough and rhinorrhea	60.0 (38.7–78.9)	88.0 (68.8–97.5)	0.74 (0.60–0.85)	5.0 (1.6–15.2)	0.5 (0.3–0.8)
**Middle**	**Peak**	Myalgia, rhinorrhea and headache	83.3 (71.5–91.7)	54.1 (46.3–61.7)	0.69 (0.62–0.75)	1.8 (1.5–2.2)	0.3 (0.2–0.6)
	**High**	Rhinorrhea, cough and headache	77.7 (71.0–83.5)	56.0 (51.1–60.8)	0.67 (0.63–0.71)	1.8 (1.5–2.0)	0.4 (0.3–0.5)
	**Low**	Cough and sore throat	61.8 (47.7–74.6)	71.6 (59.3–82.0)	0.67 (0.58–0.75)	2.2 (1.4–3.4)	0.5 (0.4–0.8)
**Old**	**Peak**	Sore throat and rhinorrhea	92.3 (74.9–99.1)	54.4 (44.8–64.1)	0.73 (0.65–0.81)	2.0 (1.6–2.6)	0.1 (0.0–0.5)
	**High**	Wheezing and rhinorrhea	77.8 (40.0–97.2)	53.0 (46.6–59.4)	0.65 (0.59–0.71)	1.7 (1.1–2.4)	0.4 (0.1–1.4)
	**Low**	Cough and rhinorrhea	76.2 (52.2–91.8)	34.8 (16.4–57.3)	0.56 (0.40–0.71)	1.2 (0.8–1.7)	0.7 (0.3–1.8)

Positive LR, Positive likelihood ratio; Negative LR, Negative likelihood ratio.

Note: Young age group (aged 18–29 years) (*n* = 307); middle age group (aged 30–65 years) (*n* = 789); old age group (aged 65+ years) (*n* = 321).

### Comparison of Clinical Manifestations in Relation to Virus Type

We compared the clinical features of patients with diagnosed with influenza A (*n* = 469) to those diagnosed with influenza B (*n* = 159). The presence of typical respiratory symptoms (rhinorrhea, cough and sore throat) was not significantly different between influenza A and B (data not shown). However, patients diagnosed with influenza B were more likely to have sore throat, nausea/vomiting, chest pain, abdominal discomfort, and diarrhea than those with influenza A (*p *< 0.05). Regarding age groups, there was no statistically significant difference in typical symptoms according to influenza virus type.

## Discussion

The recent WHO case definition of ILI, a fever of ≥38°C and a cough within seven days, has not been sufficiently accurate for use in surveillance systems and in diagnosis of influenza in previous studies [8], [9]. In fact, this definition has been used for monitoring suspected cases based on its high sensitivity rather than its specificity in accurately identifying single cases. Monto et al. suggested that fever with a cough might be a good predictor of influenza and several studies have supported the results with positive predictive values of up to 79% [5], [8], [10]–[12]. Currently, there are no gold criteria for a clinical case definition of ILI with high specificity. Several studies have reported widely variable sensitivity, specificity and predictive values of different ILI case definitions in informing the decision to treat empirically or to proceed with further testing for influenza [4], [5].

In South Korea, a community-based influenza surveillance system has been in operation, but a novel surveillance system (Hospital-based Influenza Morbidity & Mortality, HIMM) has recently been launched. HIMM relies on tertiary hospitals to monitor admission rates, morbidity and mortality related to influenza, and to collect clinical data from influenza patients. Our study demonstrated that it is impossible to obtain one single set of symptoms and signs that could distinguish influenza from various other illnesses 100% of the time. This finding is consistent with previous studies [13], [14]. We compared the accuracy of diverse case definitions (combinations of symptoms and signs) for identifying laboratory-confirmed influenza based on HIMM surveillance data during the 2011–2012 influenza season. The combinations of symptoms and signs shown in Tables 3 and 5 showed sensitivity ranging from 60% to 92% and specificity ranging from 34% to 88%. To further clarify the accuracy of ILI definitions, we analyzed the combinations in subgroups of various patient ages and different levels of influenza activity at the time of ER visit.

The accuracy of case definitions was improved by categorizing the patients into subgroups according to age. Therefore, it is useful to utilize easily obtainable demographic information to estimate influenza probability [14]. Younger patients appeared to present more classic influenza symptoms. Middle-aged patients with influenza presented more constitutional symptoms, such as headache, myalgia and typical respiratory symptoms, than those without influenza. In the oldest age group, typical respiratory symptoms were not specific for influenza patients. Among various case definitions (combinations of clinical predictors), cough and rhinorrhea in the young age group demonstrated the highest accuracy for identification of influenza, and no other combination in any other age group reached such a high level (Table 5). This result is consistent with previous studies, which showed that clinical parameters of influenza-infected older patients were relatively nonspecific [9], [15].

As the levels of influenza activity increased, the accuracy of case definitions tended to improve in this study, which is consistent with a previous finding that the clinical identification of influenza is improved when physicians are aware that influenza virus is circulating in their geographic area [16]. It has been reported that when influenza is circulating in their community, physicians can correctly diagnose influenza in more than 60%–70% of their patients on the basis of clinical symptoms alone [17]. Therefore, it is important to raise awareness in communities with increased influenza activity using the surveillance system.

Researchers previously reported that influenza A and B can infect different age groups at different rates, but may cause a similar clinical syndrome across age groups [18]. In our study population, influenza A was predominant in both the young age group and the old age group. However the ratio of influenza A to influenza B differed between the age groups and was higher in the old age group than in the young age group. Although overall symptoms of influenza A and B were similar, individuals with influenza B infection were more likely to have constitutional symptoms compared to individuals with influenza A.

There were several study limitations. The study population included only patients who fulfilled pre-specified clinical criteria (HIMM-ILI), including fever, cough, sore throat or rhinorrhea. Older patients with mild fever or without fever may be missed in this study. Also, we were not able to include patients under 18 years of age, because all the investigators participating in the surveillance system during the 2011–2012 influenza season practiced in departments that treat adult infectious diseases. In addition, as patients were recruited and enrolled at tertiary hospitals, a bias toward more severe cases, rather than those who would visit local clinics could exist. The information about comorbidities such as immunocompromised status, pregnancy, HIV/AIDS, and pregnancy that may affect the clinical presentations of influenza was not broadly available. Although analysis of data from one of the hospitals implied that comorbidities did not significantly affect the results in this study, a future study that contains those factors would be of value. As influenza A was predominant in this study population, the result could reflect the characteristics of influenza A specifically. In addition, other diagnostic tools, such as hematological testing and simple chest radiography, were not used in the evaluations, though they might augment the accuracy of case definitions [19].

The strengths of this study are that we prospectively enrolled large numbers of patients nationwide and performed laboratory diagnostic tests for influenza infection in all patients. In addition, we evaluated a broad spectrum of patients with influenza-like illness that differed in age and severity of illness. Furthermore this is the first study in Korea to evaluate the accuracy of clinical case definitions of ILI using nationwide surveillance. Future studies may validate these selected combinations of clinical variables in different seasons and populations. Also, they may estimate the accuracy in other groups and evaluate the feasibility of usage in clinical settings. A point score system, based on severity of illness, might help increase the accuracy of the case definition in diagnosing influenza [13], [20]. Finally, broader inclusion criteria would provide a more accurate combination of clinical variables with lesser selection bias. As atypical manifestations such as anorexia or altered mental status could be the only presentation of influenza, those symptoms and signs should be evaluated, especially in elderly.

For treatment guidance, in Korea, the Health Insurance Review and Assessment Service (HIRA) provides indications for antiviral agents: (1) laboratory-confirmed influenza cases, which are defined as positive for influenza with RIAT or influenza virus RT-PCR and (2) influenza-suspected cases with the physician's clinical judgment plus fever with rhinorrhea, cough or sore throat [21]. With respect to the indication of antiviral agents, the results of the present study may be useful deciding insurance coverage criteria of the government. This may be attractive as it may save laboratory costs if it is possible to identifying influenza in specific cases using only clinical symptoms, signs collected from patient history information and physical examinations.

In conclusion, this study demonstrates that the accuracy of clinical case definitions of ILI varies with patient age and level of influenza activity. This study verified that the clinical symptoms in elderly patients may be vague, and that typical influenza-like symptoms may not work well in identifying influenza more accurately. Categorizing the entire population into subgroups would improve the accuracy in identifying influenza patients in the hospital-based surveillance system.
